# Lightning Impacts on Global Forest and Carbon Dynamics: Current Understanding and Knowledge Gaps

**DOI:** 10.1111/gcb.70179

**Published:** 2025-04-07

**Authors:** Sander Veraverbeke, Thomas A. J. Janssen, Esther Brambleby, Matt Jones, Bianca Zoletto, Masha T. van der Sande

**Affiliations:** ^1^ Faculty of Science Vrije Universiteit Amsterdam Amsterdam the Netherlands; ^2^ School of Environmental Sciences University of East Anglia Norwich UK; ^3^ Plant Ecology and Nature Conservation Group Wageningen University Wageningen the Netherlands; ^4^ Forest Ecology and Forest Management Group Wageningen University Wageningen the Netherlands

**Keywords:** boreal, carbon, fire, forest, lightning, tree mortality, tropical

## Abstract

Lightning is a fundamental Earth system process that influences the world's major forest biomes and their carbon storage through two primary pathways. Lightning is the major cause of boreal forest fires, while lightning strikes kill patches of trees in tropical forests. We summarized the current understanding of these processes and identified knowledge gaps.

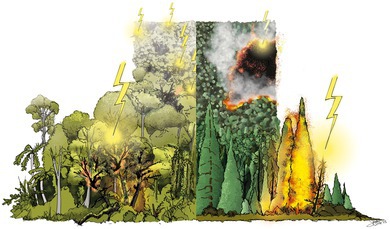

## 1 Lightning‐Induced Ecosystem Disturbance Pathways

**FIGURE 1:**
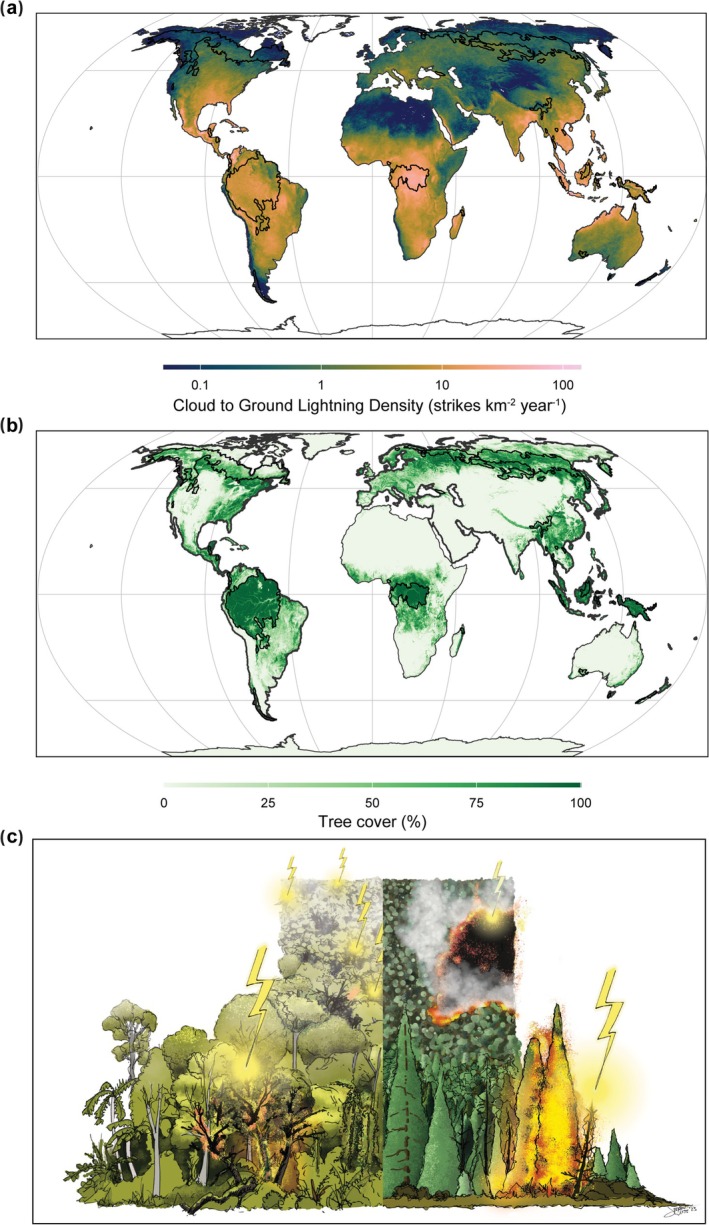
(a) Global mean annual cloud‐to‐ground lightning density overlaid with intact forest regions (black perimeters, Potapov et al. 2017). The lightning data are from the Years 2019 to 2023 and sourced from the Global Lightning Detection Network (GLD360). (b) Global fractional tree cover for the Year 2020 from Liu et al. (2024), (c) Conceptual illustration depicting patchy tropical tree mortality from abundant lightning (left) and large boreal forest fires from sparse lightning (right). Sketch by Stefan Witte.

Lightning is a widespread and fundamental Earth system process. While the drivers and patterns of global lightning distribution are well known (Kaplan and Lau 2022), its impact on the terrestrial carbon cycle remains poorly understood. Lightning affects terrestrial ecosystems and their carbon storage through two primary pathways. First, lightning ignites fires in remote forests, primarily in the seasonally dry boreal forests (Janssen et al. 2023). Although lightning strikes are relatively sparse in these areas (Figure 1), lightning‐induced fires impact large areas of boreal forest. Second, lightning can cause the death of individual trees (Gora, Burchfield, et al. 2020). This phenomenon is spatially patchy and rare in temperate and boreal forests but more prominent in tropical forests, where the world's highest lightning frequencies occur (Figure 1). In tropical forests, lightning is a major driver of large tree damage and mortality (Yanoviak et al. 2020), with potentially far‐reaching but understudied consequences for biomass turnover and carbon storage. Better understanding the role of lightning in forest dynamics and carbon storage is crucial for improving predictions of ecosystem responses to a changing climate and changing global lightning activity.

## 2 Lightning‐Induced Fires

Most lightning strikes do not ignite fires as they are often accompanied by rain during thunderstorms. Dry lightning, that is, lightning strikes with no or very little precipitation, however, is the main ignition cause of boreal forest fires. Approximately 77% of the burned area in intact extratropical forests, which are predominantly boreal forests (Figure 1), stems from lightning, while the remaining burned area has an anthropogenic cause (Janssen et al. 2023). Contemporary boreal fires emit on average 306 Tg of carbon annually, accounting for about 15% of global fire emissions (Van Wees et al. 2022). While much of this carbon emission is eventually resequestered by recovering vegetation, the ongoing increase in the frequency and severity of boreal forest fires may shift the boreal region into a net carbon source (Janssen et al. 2023; Virkkala et al. 2025).

In addition to boreal forest fires, rare Arctic tundra fires are also becoming more frequent (Descals et al. 2022). Fires in the remote tundra are almost exclusively ignited by lightning. The tundra biome and large parts of the boreal forest biome are underlain by permafrost soils, which store vast quantities of old carbon. Fires in permafrost landscapes may lead to both gradual and abrupt permafrost thaw, releasing additional greenhouse gases from decomposing soil organic matter (Natali et al. 2021). While estimates of these additional emissions remain uncertain, Natali et al. (2021) calculated that these greenhouse gas emissions could contribute up to 30% of the emissions caused by warming alone under a moderate emission scenario. While the direct carbon emissions from boreal fires are relatively well characterized (Van Wees et al. 2022), our understanding of how fires accelerate long‐term permafrost carbon losses is still in its early stages. In conclusion, although high‐latitude lightning occurs relatively infrequently during boreal summer, and only a small fraction of lightning strikes ignite fires, these large Arctic‐boreal fires have a significant and growing impact on Arctic‐boreal landscapes, particularly on forest and carbon dynamics.

## 3 Lightning‐Induced Tree Mortality

Lightning has long been thought to have a limited impact on tropical forest dynamics. However, tropical forests are global lightning hotspots, with regions such as the Congo and Amazon basins receiving up to 100 strikes per square kilometre annually (Gora, Muller‐Landau, et al. 2020, Figure 1a). Lightning affects forests not only by directly killing trees, but also by damaging nearby trees and thereby contributing to long‐term tree mortality. It has been estimated that each strike, on average, results in the death of 3.5 trees and damages an additional 11.4 trees (Gora, Burchfield, et al. 2020). In tropical forests, lightning is estimated to damage more than 800 million trees and kill approximately 200 million trees annually. Furthermore, lightning strikes disproportionally kill and damage the largest trees within a forest patch (Gora, Burchfield, et al. 2020; Yanoviak et al. 2020). Given the crucial role of large trees in carbon storage, biodiversity conservation, and seed production, lightning can significantly influence key ecosystem processes in tropical forests. Indeed, Gora, Burchfield, et al. (2020) found that lightning frequency is an even stronger predictor of variation in aboveground biomass storage and turnover than key environmental drivers such as precipitation or temperature.

Despite the critical role of lightning in tropical forests, our understanding of its effects remains limited. For instance, it is still unknown how and why species differ in their resistance to lightning‐caused damage. For example, tree species that grow very tall are generally more likely to be struck by lightning and would therefore be expected to evolve a high resistance to lightning damage. If species do indeed vary in their resistance to lightning damage, then lightning frequency may have acted as a selective pressure over time, influencing both species composition and the carbon cycle of tropical forests. Furthermore, although lightning strikes in tropical forests rarely ignite forest fires (Janssen et al. 2023), the possible increase in lightning frequency combined with more frequent drought events may increase the likelihood of lightning‐induced tropical forest fires. Historically, fires have been largely absent from wet tropical forests, leaving them particularly vulnerable to fire‐induced tree mortality. Hence, lightning in tropical forests is a major cause of tree mortality and may, if becoming a more important ignition source of tropical forest fires, result in significant biodiversity and carbon losses.

## 4 Knowledge Gaps and Directions

Our perspective synthesizes two key impacts of lightning on terrestrial carbon dynamics in the two major remaining forest biomes on Earth, through boreal forest fires and lightning‐induced tropical tree mortality. Furthermore, we highlight the importance of unprecedented lightning‐ignited fires in the Arctic tundra, which may be particularly vulnerable to fire‐induced permafrost thaw. We argue that the impact of lightning on these critical ecosystems and their carbon dynamics is currently understudied and the processes involved are not adequately characterized or quantified to be included in Earth system models (ESMs). A major uncertainty lies in the fate of lightning in a warmer world. Different lightning parametrizations yield diverging predictions of future lightning density over the boreal and tropical forest biomes. While all predictions suggest an increase in lightning over boreal forests due to climate change, the magnitudes of this increase vary (Janssen et al. 2023). Over tropical forests, in contrast, future lightning predictions using different model parametrizations diverge in sign. This uncertainty in future lightning activity introduces a significant constraint on modeling future impacts of lightning on tropical tree mortality and fires.

Few fire‐enabled ESMs and vegetation models explicitly incorporate the spatial and temporal patterns of anthropogenic and lightning ignitions on Earth, along with their distinct drivers. We argue that a spatially explicit approach to fire cause attribution is necessary to determine how future global changes will impact the probability of both lightning‐ and human‐ignited fires, as well as their impacts on carbon dynamics. This includes the impact of fire on permafrost thaw and associated emissions. Because of our limited understanding of fire–permafrost interactions, no current ESMs routinely include this interaction. As a result, the impact of lightning on boreal forest and permafrost carbon dynamics through fire remains poorly understood.

Lightning is a major cause of tree damage and mortality across the tropics, and its future role as an ignition source of tropical forest fires remains unknown. We have limited understanding of the tropical forest's resistance to lightning fires and its capacity to recover from such disturbances. Furthermore, the extent to which tree species possess adaptations to survive lightning damage is currently unknown. Understanding the impacts of lightning, both directly through tree mortality and indirectly through fire ignition, will be crucial to minimize fire risks, enhance forest recovery, and protecting the world's largest terrestrial carbon stocks.

## Author Contributions

**Sander Veraverbeke:** conceptualization, data curation, funding acquisition, project administration, writing – original draft. **Thomas A. J. Janssen:** formal analysis, writing – review and editing. **Esther Brambleby:** data curation, writing – review and editing. **Matt Jones:** data curation, writing – review and editing. **Bianca Zoletto:** writing – original draft, writing – review and editing. **Masha T. van der Sande:** writing – original draft, writing – review and editing.

## Conflicts of Interest

The authors declare no conflicts of interest.

## Data Availability

The tree cover and intact forest data in Figure 1 are publicly available. The lightning data in Figure 1 can be obtained from the corresponding author upon request.
